# Temporal Dynamics of tsRNA Regulation Mark an Abrupt Transition After Epileptogenesis

**DOI:** 10.1111/jnc.70317

**Published:** 2025-12-15

**Authors:** Saad Zaheer, Sharada Baindoor, Niamh M. C. Connolly, Kai Siebenbrodt, Sebastian Bauer, Felix Rosenow, Jens S. Andersen, Morten T. Venø, Jørgen Kjems, David C. Henshall, Jochen H. M. Prehn

**Affiliations:** ^1^ Department of Physiology & Medical Physics and RCSI Centre for Systems Medicine RCSI University of Medicine and Health Sciences Dublin Ireland; ^2^ FutureNeuro Research Ireland Centre RCSI University of Medicine and Health Sciences Dublin Ireland; ^3^ Goethe‐University Frankfurt, Epilepsy Center Frankfurt Rhine‐Main, Department of Neurology University Medical Center Frankfurt Germany; ^4^ Center for Personalized Translational Epilepsy Research (CePTER) Goethe‐University Frankfurt Frankfurt Germany; ^5^ Center for Experimental Bioinformatics University of Southern Denmark Odense M Denmark; ^6^ Omiics ApS Aarhus Denmark; ^7^ Interdisciplinary Nanoscience Center, Dept. of Molecular Biology and Genetics Aarhus University Aarhus Denmark

**Keywords:** Ago2‐immunoprecipitation, multi‐omics integration, temporal lobe epilepsy, tRNA‐derived small RNAs

## Abstract

Epileptogenesis involves widespread molecular remodeling, including transcriptional and post‐transcriptional changes that reshape neuronal networks. While microRNAs have been extensively studied in this context, the contribution of transfer RNA‐derived small RNAs (tsRNAs) remains largely unexplored. Understanding how tsRNAs engage in Argonaute 2 (Ago2)‐mediated regulation during epileptogenesis could uncover new layers of post‐transcriptional control relevant to seizure development and progression. Recent studies increasingly recognize transfer RNA‐derived small RNAs or tsRNAs, especially those bound to Argonaute 2 (Ago2), as functional regulators of gene expression. Here, we analyzed Ago2‐immunoprecipitated small RNA‐Seq data along with matching transcriptomic and proteomic data across seven defined timepoints in a rat model of epilepsy that was induced using perforant pathway stimulation (PPS). The analysis showed dynamic shifts in Ago‐2 bound tsRNA expression, with early and intermediate stages showing upregulation of shorter tsRNA fragments, whereas Day of First Seizure (DOFS) and chronic timepoints showed a shift toward 5′ tiRNAs, including highly upregulated GlyGCC‐derived fragments. Cluster analysis using Weighted Gene Co‐Expression Network Analysis (WGCNA) identified modules specific to the DOFS timepoint where tsRNAs clustered together with genes enriched in pathways including neuronal metabolism, mitochondrial function, and synaptic stability. Target prediction analysis using RNAhybrid at DOFS predicted targets in 3′ UTR, 5′ UTR, and CDS regions showing an association with glycolysis, protein localization, and vesicle trafficking. Subsequent gene‐disease association analysis further associated the predicted targets with neurodegenerative conditions including but not limited to Alzheimer's disease, intellectual disability, and epilepsy. This study highlights that tsRNAs potentially play a temporal dynamic regulatory role in epileptogenesis with an evident shift in tsRNA accumulation at DOFS suggesting a potential rewiring of post‐transcriptional control at the completion of epileptogenesis. This work also highlights a first integrative approach of tsRNA downstream effects on the transcriptome and proteome in epilepsy and suggests innovative tsRNA‐driven mechanisms relevant to disease progression.

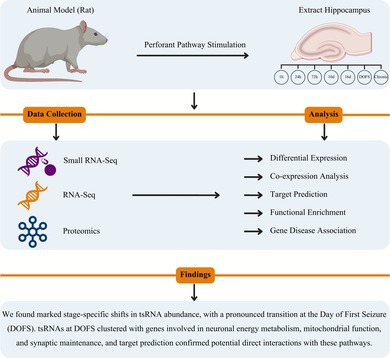

## Graphical abstract

Perforant pathway stimulation was used to induce epilepsy in rats to investigate temporal regulation by tRNA‐derived small RNAs (tsRNAs). Across key stages of epileptogenesis, Ago2‐bound small RNA‐Seq, transcriptomics, and proteomics were integrated. We found marked stage‐specific shifts in tsRNA abundance, with a pronounced transition at the Day of First Seizure (DOFS). tsRNAs at DOFS clustered with genes involved in neuronal energy metabolism, mitochondrial function, and synaptic maintenance, and target prediction confirmed potential direct interactions with these pathways. These findings reveal a dynamic, seizure‐associated regulatory role for tsRNAs and highlight their potential influence on epileptogenic progression.

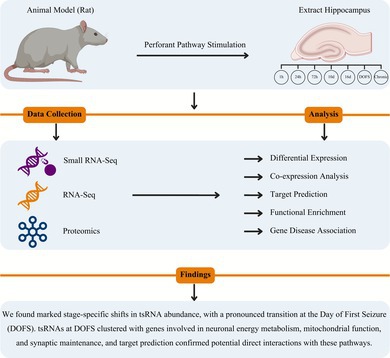

**Abbreviations**
Ago: argonaute
Ago2: argonaute 2
Ago2‐IPed: Ago2‐immunoprecipitated
ARRIVE: animal research: reporting of in vivo experiments
ATP: adenosine triphosphate
CDS: coding sequence
DE: differential expression
DEGs: differentially expressed genes
DEPs: differentially expressed proteins
DETs: differentially expressed tsRNAs
DNA: deoxyribonucleic acid
DOFS: day of first seizure
Easy‐nLC: easy nano liquid chromatography
EEG: electroencephalography
FC: fold change
FDR: false discovery rate
GO: gene ontology
GO BP: gene ontology biological process
HCD: higher‐energy collisional dissociation
IP: immunoprecipitation
KEGG: Kyoto Encyclopedia of Genes and Genomes
kME: module eigengene‐based connectivity (correlation between a gene and its module eigengene)
LC–MS: liquid chromatography–mass spectrometry
MFE: minimum free energy
miRNA: microRNA
mRNA: messenger RNA
MS: mass spectrometry
MS2: tandem mass spectrometry (MS/MS)
NaCl: sodium chloride
ncRNA: non‐coding RNA
PPS: perforant pathway stimulation
pre‐miRNA: precursor microRNA
PRIDE: proteomics identifications database
pri‐miRNA: primary microRNA
Q Exactive HF: Q exactive HF hybrid quadrupole‐orbitrap mass spectrometer
RIP‐Seq: RNA immunoprecipitation sequencing
RISC: RNA‐induced silencing complex
RNA: ribonucleic acid
RNA‐Seq: RNA sequencing
SD: standard deviation
sncRNA: small non‐coding RNA
tiRNA: tRNA half (tRNA‐derived stress‐induced RNA)
TLE: temporal lobe epilepsy
tRF: tRNA‐derived fragment
tRNA: transfer RNA
tsRNA: tRNA‐derived small RNA
UTR: untranslated region
VST: variance‐stabilizing transformation
WGCNA: weighted gene co‐expression network analysis

## 1 Introduction

Temporal lobe epilepsy (TLE) is a chronic neurological disorder characterized by recurrent seizures originating in temporal lobe structures (hippocampus, amygdala, temporal cortex). Analysis of surgically obtained brain samples from TLE patients consistently finds patterns of selective neuron loss as well as gliosis, inflammatory markers, and local and distant changes to neuronal network connections (Blumcke et al. 2017). Gene expression profiling studies have identified dysregulation of the protein‐coding genes that serve these functions (Johnson et al. 2015; Pitkänen and Lukasiuk 2011). Both the neuropathology and gene expression changes can be modeled in rodents, including by locally applied chemoconvulsants (e.g., kainic acid) (Conte et al. 2020; Rusina et al. 2021) or repetitive electrical stimulation of afferent pathway fibers to the hippocampus (Khemka et al. 2024; Sloviter 2024). Improvements in our understanding of the causal pathomechanisms may generate insights for molecular biomarkers of the process of epileptogenesis and transition to the chronic epileptic state, as well as new therapeutic targets for drug‐resistant epilepsy (Delahaye‐Duriez et al. 2016; Klein et al. 2024).

Small non‐coding RNAs (sncRNAs) are important regulators of gene expression and protein translation, among many other functions (Patil et al. 2014). The best characterized group of sncRNAs is microRNA (miRNA), sncRNAs of 21–25 nucleotides in length that function post‐transcriptionally to negatively regulate protein levels via sequence‐specific binding to the 3′ untranslated region (UTR) of target mRNAs (E. C. Lai 2002). This process promotes either mRNA degradation or translational repression. miRNAs are transcribed from DNA as initially longer sequences called ‘pri‐miRNA’, which are then processed in the nucleus by Drosha to generate ‘precursor miRNA’ (pre‐miRNA) of around 70 nucleotides in length. Pre‐miRNAs are exported from the nucleus and processed in the cytoplasm by the nuclease, Dicer (Ha and Kim 2014). Processing by Dicer generates double‐stranded RNA duplexes. Next, one strand, the guide, is loaded into the binding pocket of an argonaute (Ago) protein to form an RNA‐induced silencing complex (RISC). Thereafter, the RISC identifies ‘seed’ matches between the 5′ end of the miRNA and the 3′ UTRs of mRNAs, resulting in reduced protein levels of the target mRNAs (Gebert and MacRae 2019).

Recent studies have identified another important source of small non‐coding RNAs, transfer RNA‐derived small RNAs (tsRNA). These also have the capacity to regulate gene expression and protein translation (Schimmel 2018; Su et al. 2020; Suzuki 2021; Winek and Soreq 2025). tsRNAs derive from mature tRNAs and are enriched in many tissues including the nervous system (Fagan et al. 2021; Winek and Soreq 2025), where they are often more abundant than miRNA (Baindoor et al. 2024; Jehn et al. 2020; Mesquita‐Ribeiro et al. 2021). Cleavage of tRNA can occur in the anti‐codon loop through the stress‐induced ribonuclease, Angiogenin. Cleavage in the anti‐codon loop generates 5′ and 3′ tRNA‐derived stress induced RNAs (tiRNAs) of 31–40 nucleotides in length. tiRNAs have been shown to regulate stress granule formation, protein translation, and gene expression (Emara et al. 2010; Schimmel 2018; Su et al. 2020). Interestingly, tsRNA can also be generated by the activity of Dicer. Dicer cleavage generates smaller fragments of 14–30 nucleotides in length as this cleavage occurs within the D‐ or T‐loop of tRNAs, generating either 5′ tRNA‐derived fragments (tRFs) or 3′ tRFs, respectively (Baindoor et al. 2024; Cole et al. 2009; Winek and Soreq 2025). In further analogy to miRNAs, tRFs and tiRNA have been shown to bind to Ago2 and other members of the Ago family (Azuma‐Mukai et al. 2008; H. Lai et al. 2023; Winek et al. 2020). Furthermore, 3′ tRFs have been shown to function in a pathway identical or similar to that of miRNA (Kuscu et al. 2018).

We recently identified elevated levels of several 5′ tRFs in the plasma of TLE patients in advance of epileptic seizures (Hogg et al. 2019). However, their temporal association with the process of epileptogenesis, miRNA expression changes, gene expression, and alteration in protein levels has not yet been explored. Here, we provide a comprehensive analysis of Ago2‐bound tsRNAs during the progression of epileptogenesis in the established rodent model of TLE induced by perforant path stimulation (Gomes‐Duarte et al. 2021; Khemka et al. 2024; Venø et al. 2020), and conduct an integrated analysis of tsRNA production in relation to transcriptome and proteome changes. We identified a marked collapse in shorter tsRNA expression at the onset of spontaneous seizures, coinciding with sharp transcriptomic and proteomic shifts. These findings suggest that tsRNAs may play a critical regulatory role in driving molecular transitions that shape the disease trajectory in temporal lobe epilepsy.

## 2 Methods

### 2.1 Experimental Model and Data Design

The datasets analyzed in this study were obtained from a previously published investigation of epileptogenesis using the perforant pathway stimulation (PPS) model of TLE in rats (Venø et al. 2020). In that study, Argonaute‐2‐immunoprecipitated (Ago2‐IP) small RNA‐Seq, bulk RNA‐Seq, and quantitative proteomics were generated from hippocampal tissue collected at seven defined stages after PPS, enabling matched multi‐omics analysis of molecular changes during epileptogenesis.

### 2.2 Animals

Adult male Sprague–Dawley rats (age 2–6 months; typical body weight 325–350 g at the time of stimulation) were used. Animals were sourced primarily from Charles River Laboratories (Germany). Harlan Laboratories served as the secondary supplier when delivery issues occurred. All relevant procedures complied with EU Directive 2010/63/EU, ARRIVE guidelines, the regional ethics approval from the Regierungspräsidium Giessen, Germany (approval number 73/2013), or were carried out under the Animals (Scientific Procedures) Act 1986 (United Kingdom).

### 2.3 Housing and Husbandry

Prior to PPS surgery, rats were housed in plastic “Type 4” cages (55 × 33 × 20 cm, Ehret, Germany) with matching mesh lids (“Type 4 Standard”; 55.7 × 33.2 × 0.5 cm) in groups of 2–6 animals. After electrode implantation, animals were housed individually in double‐height Type 3 plastic cages (in‐house production) but were always maintained with visual and auditory contact with at least one conspecific.

Housing rooms were maintained at 22°C, 55%–60% humidity, and a 12:12 light–dark cycle. Water (acidified to pH 3.0–3.5) and standard rodent chow were provided ad libitum. Softwood pellets were used as bedding.

### 2.4 PPS Procedure and Experimental Timepoints

Unilateral PPS was performed under inhaled isoflurane anesthesia (5% induction; 2%–3% maintenance). Buprenorphine (0.2 mg/kg, s.c.) was administered for perioperative analgesia. Following a 1‐week recovery, PPS was applied as previously described by Venø et al. (2020) to induce status epilepticus without causing self‐sustaining seizures or mortality. Continuous video‐EEG monitoring was performed for up to 3 months. Hippocampi were collected at seven timepoints representing epileptogenesis and early epilepsy: 1 h, 24 h, 72 h, 10 days, 16 days, day of first seizure (DOFS), and chronic epilepsy (≈1 month after first spontaneous seizure). Rats were euthanized under deep anesthesia (xylazine + ketamine) by transcardial perfusion with ice‐cold 0.9% NaCl.

### 2.5 Controls and Replication

**FIGURE 1:**
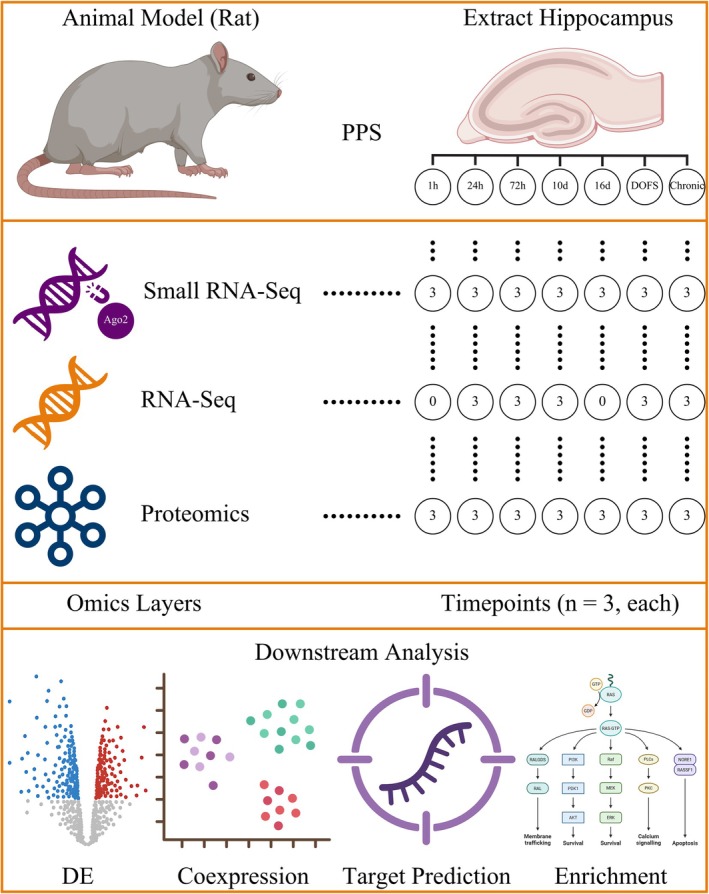
Overview of experimental model and multi‐omics data collection. Epilepsy was introduced using perforant pathway stimulation (PPS) in adult rats and hippocampal tissue samples were collected at seven timepoints after stimulation: 1 h, 24 h, 72 h, 10 days, 16 days, day of first seizure (DOFS), and chronic epilepsy. For each PPS‐exposed timepoint, *n* = 3 rats were analyzed. A single shared control group (*n* = 3 rats), collected at the 10‐day timepoint, was used as the reference for all omics comparisons throughout the study. These same three control animals were processed across all platforms and are shown alongside each PPS‐exposed timepoint for differential analyses. No timepoint‐specific control groups were generated. Matched datasets were generated from the same cohort of animals across three omics layers: (1) Argonaute‐2‐immunoprecipitated (Ago2‐IP) small RNA sequencing. (2) RNA sequencing (RNA‐Seq), and (3) Tandem mass‐tagged quantitative proteomics. The lower panel outlines the major downstream analytical steps including differential expression analysis, weighted gene co‐expression network analysis (WGCNA), target prediction, and functional pathway enrichment.

A single cohort of sham‐stimulated control rats (*n* = 3), collected at 10 days post‐surgery, was used as the shared reference group for all PPS‐exposed timepoints and across all omics platforms. This design matches the original (Venø et al. 2020) dataset and ensures consistency across small RNA‐Seq, RNA‐Seq, and proteomics. Each PPS‐exposed timepoint included *n* = 3 rats, except RNA‐Seq, which did not include samples at 1 h or 16 days. The sequencing datasets are available on Gene Expression Omnibus GSE137473, and the proteomics data are available on Proteomics Identifications Database (PRIDE) PXD019098 (Figure 1).

### 2.6 Omics Library Preparation and Data Generation

**Small RNA‐Seq:** The original study (Venø et al. 2020) describes the protocols for tissue collection, RNA extraction, Ago2 immunoprecipitation, library preparation, and sequencing in detail. Briefly, Ago2‐immunoprecipitated small RNA‐Seq (RNA Immunoprecipitation Sequencing or RIP‐seq) was performed from hippocampal lysates to capture functionally active small RNAs, including miRNAs and transfer RNA‐derived small RNAs (tsRNAs). **RNA‐Seq:** Total RNA was extracted from hippocampal tissue and subsequently subjected to ribosomal RNA depletion using Ribo‐Zero Magnetic Kit (Illumina) to enrich for mRNAs and non‐rRNA species. ScriptSeq v2 kit (Illumina) was used to prepare sequencing libraries, and Agilent 2100 Bioanalyzer was used to assess library quality. Finally, Illumina HiSeq platform was used to sequence libraries, generating high‐throughput paired‐end reads. While our analytical pipeline differs from the original setup (see RNA‐Seq analysis), library preparation and sequencing were performed as described in the original study (Venø et al. 2020). **Proteomics:** Frozen hippocampal tissues were lysed in a guanidine‐based buffer with protease and phosphatase inhibitors, sonicated, and clarified using centrifugation. Proteins were reduced, alkylated, and digested using LysC and trypsin. TMTsixplex isobaric labelling reagents, from Thermo Fisher Scientific, were used to label peptides, and liquid chromatography‐mass spectrometry (LC–MS) confirmed the labelling efficiency. Peptides were fractionated by high‐pH reverse‐phase chromatography using custom‐packed StageTips and eluted across 17 gradient steps. A Q Exactive HF mass spectrometer coupled to an Easy‐nLC system was used to analyze samples using a 120‐min gradient and data‐dependent acquisition mode (Top12). Higher‐energy collisional dissociation (HCD) was employed by tandem mass spectrometry (MS/MS). MaxQuant (v1.5.3.30) with Andromeda as search engine was used to process the data using the rat UniProt database (August 2016). Isobaric label quantification was performed using the reported ion MS2 method with normalization to a common timepoint and internal control to derive relative protein abundance.

#### 2.6.1 Small RNA‐Seq Analysis

tsRNAsearch, a publicly available and validated Nextflow pipeline (Donovan et al. 2021), was used to identify and quantify tsRNAs in the hippocampus across different stages of epileptogenesis. Raw FASTQ files were passed as inputs to the pipeline. The pipeline includes adapter trimming using standard trimming tools to remove sequencing adaptors, alignment to a custom non‐coding RNA (ncRNA) reference optimized for tRNA‐derived fragments mapping, reads collapsing to generate count‐level summaries across all mapped reads for each tsRNA species, and generation of depth files utilizing four integrated methods to quantify nucleotide‐level coverage across tRNA sequences. These steps minimize false positives and provide accurate read distribution across canonical tRNA regions, for example, 3′ tRFs or 5′ tiRNAs. While the tsRNAsearch pipeline is fully capable of performing statistical testing for differential expression using Fisher's method, we chose to stop at the read count matrix output and process it independently.

Differential expression analysis of tsRNAs was performed using DESeq2 (Love et al. 2014). Read counts were normalized using DESeq2's median‐of‐ratios method, and statistical significance was determined using the Benjamini–Hochberg FDR method to adjust for multiple testing, with a threshold of adjusted *p*‐value < 0.05 and |log2FC| ≥ 0.5 to define differentially expressed tsRNA (DETs).

#### 2.6.2 RNA‐Seq Analysis

Raw RNA‐Seq data were obtained from Venø et al. (2020). Quality control and preprocessing were performed. FastQC was used to assess read quality, and Trimmomatic (Bolger et al. 2014) was used to trim adapter sequences and low‐quality bases. A second quality control check using FastQC was performed to confirm the removal of adapter sequences and ensure high‐quality reads for downstream analysis.

Kallisto, a pseudoalignment‐based quantification tool that enables rapid and accurate transcript abundance estimation (Bray et al. 2016), was used to process the high‐quality reads. Ensembl (Dyer et al. 2025) mRatBN7.2 rat reference transcriptome was used to build a transcriptome index. Finally, quantification was performed using default parameters, yielding both transcripts per million (TPM) and estimated read counts.

DESeq2 was used to perform differential gene expression analysis using the count data generated by kallisto. Tximport (Soneson et al. 2015) was used to summarize transcript‐level counts to gene‐level counts prior to analysis. Size factors were estimated and log2 fold changes were calculated using DESeq2. A threshold of adjusted *p*‐value (Benjamini–Hochberg FDR) < 0.05 and |log2FC| ≥ 0.5 was used to define differentially expressed genes (DEGs) at each timepoint compared to control samples.

#### 2.6.3 Proteomic Analysis

Quantitative proteomics data were obtained from Venø et al. (2020) and processed using the same workflow described therein. In short, protein lysates from hippocampal tissue samples were digested and subjected to label‐free liquid chromatography mass spectrometry (LC–MS/MS). A rat‐specific Uniprot database was used to perform protein identification and quantification.

Following normalization, two complementary approaches, including Limma, a linear modeling approach designed for small sample sizes, and Rank Product analysis, a non‐parametric method that highlights consistently up‐ or down‐regulated proteins across replicates, were used to assess differential protein expression.

Using both methods, proteins with statistically significant expression changes, that is, *q*‐value < 0.05 and |log2FC| ≥ 0.5 were considered differentially expressed proteins (DEPs).

### 2.7 Weighted Gene Co‐Expression Network Analysis (WGCNA)

To identify groups of genes and tsRNAs with coordinated expression patterns across disease stages, we performed WGCNA (Langfelder and Horvath 2008) using matched RNA‐Seq and small RNA‐Seq datasets. Expression data from the five overlapping timepoints (24 h, 72 h, 10 days, DOFS, and chronic) were combined to generate pooled raw count matrices. Shared control samples were used as the baseline reference for all PPS‐exposed timepoints.

The pooled raw counts were variance‐stabilized using the vst() function from the DESeq2 package for R programming language to normalize for sequencing depth and variance heterogeneity. A signed co‐expression network was then constructed using WGCNA, followed by hierarchical clustering to detect modules (clusters) of co‐expressed genes and tsRNAs.

To identify biologically meaningful modules, we calculated module‐trait correlations, which quantify the strength of association between each module's eigengene (its first principal component) and experimental conditions (PPS‐exposed vs. control at each timepoint). Since WGCNA requires numeric variables, categorical timepoints were encoded as binary vectors (e.g., DOFS samples = 1, control samples = 0). Modules showing strong correlations (|*r*| ≥ 0.95, *p* < 0.01) with specific timepoints were retained for downstream analyses.

With each selected module, we identified hub genes and tsRNAs, those showing high intramodular connectivity (|kME| > 0.8), as the most central and potentially functionally important members of each network.

### 2.8 Functional Enrichment Analysis

To identify overrepresented biological processes and pathways among gene sets of interest, Gene ontology (GO) and Kyoto Encyclopedia of Genes and Genomes (KEGG) enrichment analyses were conducted using clusterProfiler (Yu et al. 2012).

Gene sets were checked against the GO biological process (BP) ontology and KEGG pathway databases using enrichGO() and enrichKEGG() functions in the R programming language. Benjamini–Hochberg adjusted FDR *p*‐value < 0.05 was used to filter enrichment results and subsequently visualized as dot plots.

### 2.9 tsRNA Target Prediction and Disease Enrichment

We performed target prediction using RNAhybrid (v2.1.2) (Krüger and Rehmsmeier 2006) to investigate potential regulatory interactions between tsRNAs and mRNAs. We used DETs identified at DOFS as query sequences. Putative mRNA targets were identified among DEGs and DEPs (mapped to gene symbols) at the DOFS timepoint. 5′ UTR, 3′ UTR, and CDS sequences for these genes were retrieved using BioMart (Ensembl, *Rattus norvegicus* genome) (Durinck et al. 2009). RNAhybrid was used to predict hybridization between tsRNA and mRNA sequences for each region. Predicted interactions with minimum free energy (MFE) ≤ −20 kcal/mol and *p*‐value < 0.05 were considered significant.

To further explore the relevance of the predicted targets, we performed GO BP enrichment analysis as described above. To also check for disease relevance, the predicted target genes were mapped to human orthologs using Rat Genome Database (Vedi et al. 2023) and submitted to DisGeNET (Piñero et al. 2017). Only curated gene‐disease associations with a DisGeNET score ≥ 0.8 were retained and finally visualized using a disease enrichment plot using the disgenet2r package in R programming language.

## 3 Results

### 3.1 Temporal Remodeling of Ago2‐Associated tsRNAs Reveals Stage‐Specific Shifts in Post‐Transcriptional Regulation

Utilizing an established Ago2‐immunoprecipitated (Ago2‐IPed) small RNA‐Seq dataset obtained from hippocampal tissue of PPS‐exposed and control rats (Venø et al. 2020), we investigated all small non‐coding RNAs (sncRNAs) associated with Ago2 protein to examine the role of tsRNAs in the post‐transcriptional regulatory mechanisms underlying epileptogenesis in a rodent model of TLE. To find out whether Ago2‐bound small RNAs exhibit temporal regulatory changes after PPS exposure, we first profiled the abundance, length, and composition of sncRNAs across key timepoints of epileptogenesis. This allowed us to study dynamic changes in Ago2‐bound small RNAs, especially tsRNAs.

**FIGURE 2:**
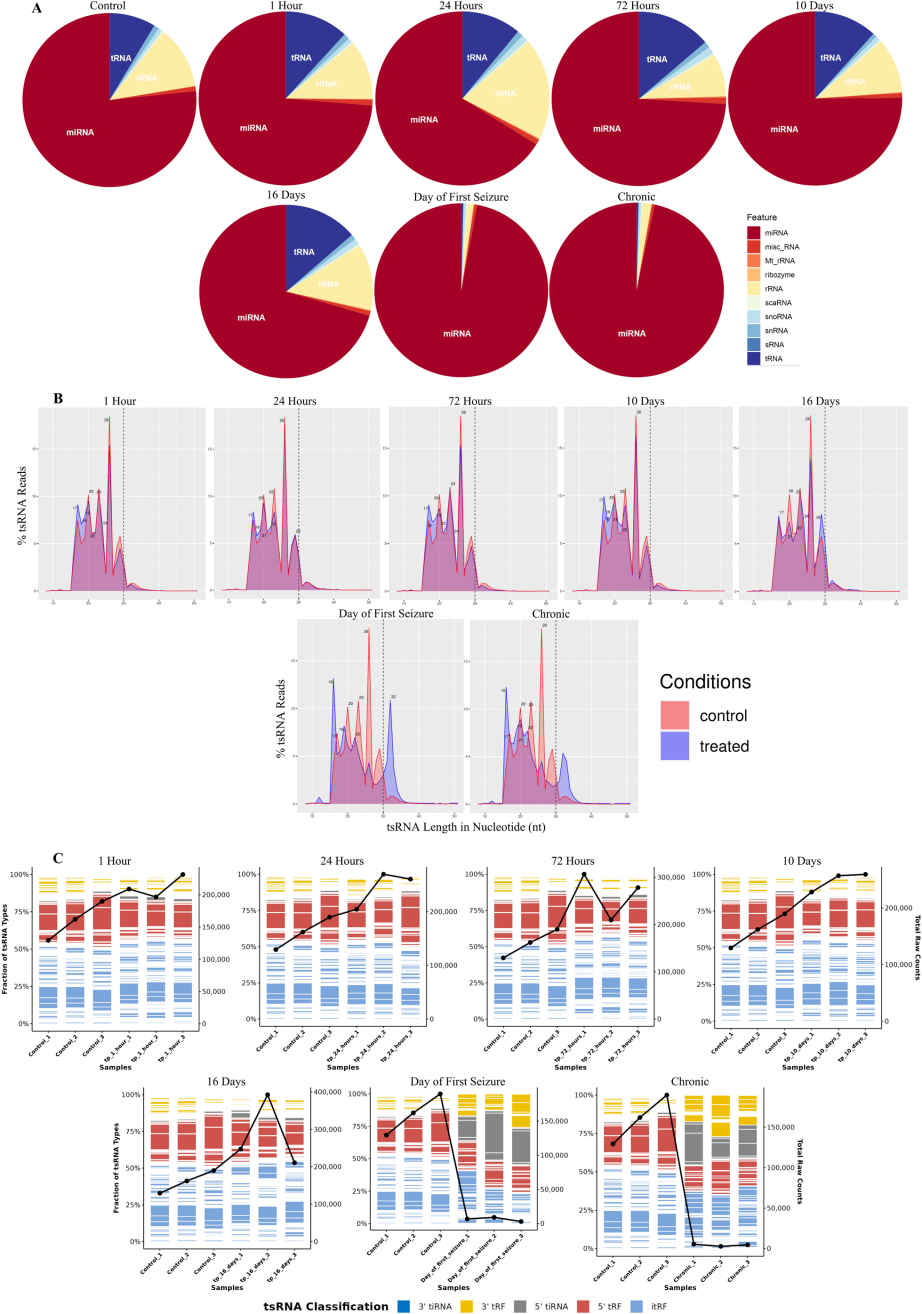
Temporal dynamics of Ago2‐bound small RNA classes, length distribution, and tsRNA abundance. (A) Venn diagrams showing proportions of major small non‐coding RNA (sncRNA) classes captured by Argonaute‐2 (Ago2) immunoprecipitation in control (*n* = 3 rats) and Perforant Pathway Stimulation (PPS)‐exposed hippocampal samples across seven timepoints (*n* = 3 rats per group). While microRNAs (miRNAs) dominate across all conditions, PPS‐exposed samples at the Day of First Seizure (DOFS) and chronic timepoints show a reduction in the overall transfer RNA (tRNA)‐derived reads and a corresponding increase in miRNAs. (B) Density plots showing the distribution of tRNA‐derived small RNA (tsRNA) lengths in control (pink) and PPS‐exposed (labeled “Treated” and blue) samples at each timepoint (*n* = 3 rats per group). tsRNAs at early timepoints are primarily < 30 nucleotides, but a shift toward longer fragments (30–40 nt) is observed at DOFS and Chronic stages in the PPS‐exposed samples. (C) Stacked bar plot showing the relative composition (left *y*‐axis) and total abundance (black line, right *y*‐axis) of tsRNAs in shared control (*n* = 3 rats) and PPS‐exposed samples across timepoints (*n* = 3 rats per group). The same cohort of control samples (*n* = 3 rats) was used for all comparisons. Early and intermediate timepoints exhibit increased tsRNA abundance in PPS‐exposed samples relative to shared controls, whereas DOFS and chronic timepoints show a sharp reduction.

Across all timepoints, miRNAs were the predominant Ago2‐bound sncRNA species followed by tRNA‐ and rRNA‐derived reads (Figure 2A). While this pattern remained relatively consistent in controls and PPS‐exposed timepoints, PPS‐exposed samples at the day of first seizure (DOFS) and chronic stages showed a marked decrease in tRNA reads and a proportional increase in miRNAs. This shift in composition may reflect selective recruitment of miRNAs over tsRNAs into Ago2 complexes after epileptogenesis.

We next studied the length distributions of Ago2‐bound tsRNAs as these may reflect differences in biogenesis or functional class, for example, tRFs versus tiRNAs. In both groups of samples, that is, Control and PPS‐exposed, tsRNAs predominantly fell under 30 nucleotides, which is typical of tRFs. However, at DOFS and chronic timepoints, PPS‐exposed samples showed a shift toward longer fragments (30–40 nt) (Figure 2B). This shift is consistent with increased tiRNA accumulation, which has been linked to cellular stress and translational repression.

To further understand the dynamics of tsRNA abundance and subtype usage, we studied their relative compositions and total read content across timepoints (Figure 2C). Notably, at early and intermediate stages, total tsRNA abundance in PPS‐exposed samples increased compared to the shared control samples. This possibly reflects an early adaptive or compensatory response. However, at DOFS and chronic stages, the abundance of tsRNAs declined sharply. This suggests a collapse of regulatory plasticity or a shift toward other post‐transcriptional regulators such as miRNAs.

**FIGURE 3:**
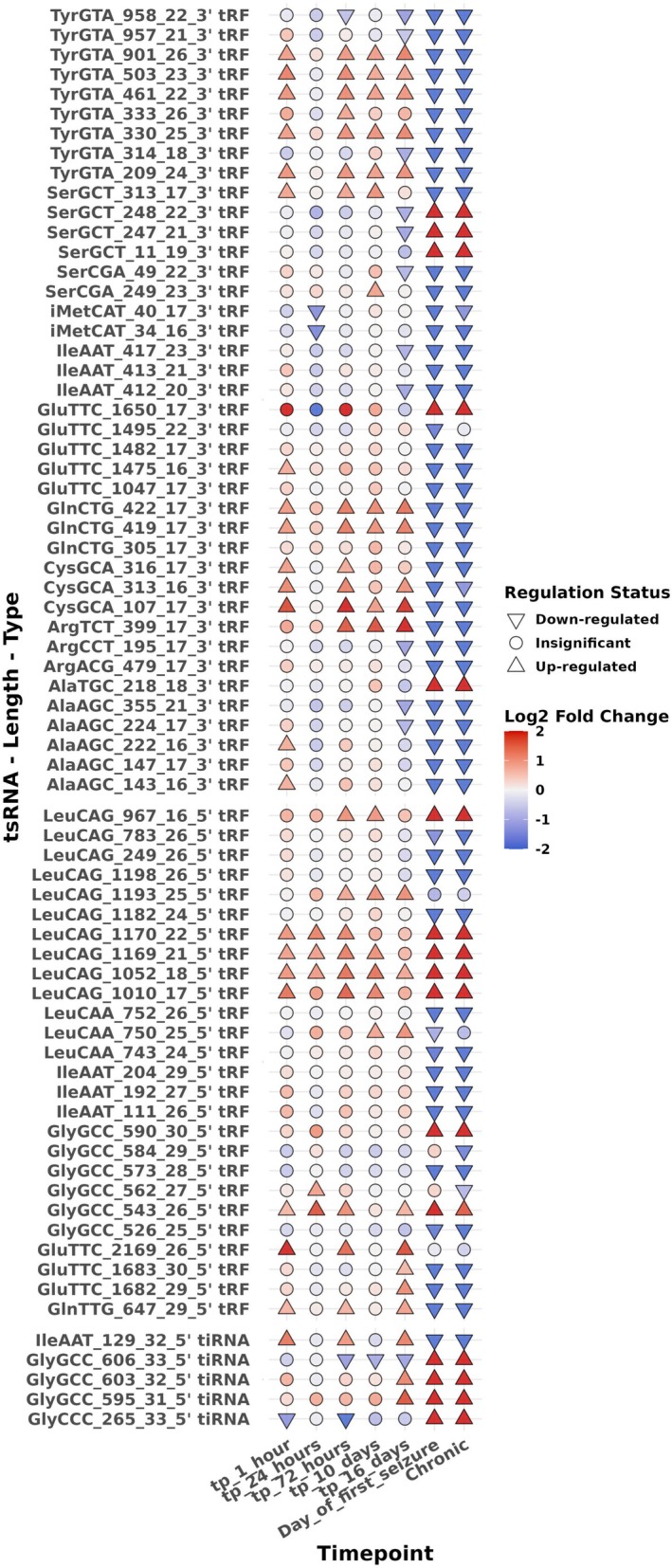
Bubble plot summarizing differential expression of individual tsRNAs across timepoints. Each row represents a transfer RNA‐derived small RNA (tsRNA) species. Color intensity indicates fold‐change magnitude, and shape indicates direction (triangles = up/downregulated; circle = not significant). All comparisons represent perforant pathway stimulation (PPS)‐exposed samples (*n* = 3 rats) versus the shared control group (*n* = 3 rats). Early stages show moderate upregulation, whereas the Day of First Seizure (DOFS) and chronic stages show noticeable changes, including upregulation of 5′ tRNA half or 5′ tRNA‐derived stress‐induced RNA (5′ tiRNA) GlyGCC fragments and downregulation of 5′ tiRNA IleAAT.

We finally examined the differential expression patterns of all Ago2‐bound tsRNAs at each timepoint to explore the dynamic regulation of individual tsRNA isoacceptors across epileptogenesis (Figure 3). To visualize temporal regulation trends, each tsRNA species was annotated with its isoacceptor identity and subtype, for example, 3′ tRF, 3′ tiRNA, 5′ tRF, or 5′ tiRNA. Early timepoints showed an upregulation trend, whereas DOFS and chronic timepoints showed more dramatic expression changes, especially among tiRNAs. For example, the 5′ tiRNA GlyGCC was stable early on but strongly upregulated at later stages, whereas the 5′ tiRNA IleAAT showed an opposite trend. These trends suggest that tsRNAs may act as stage‐specific regulators, with some species activated in response to acute insults and others emerging later on during the transition to final chronic disease states.

Together, these findings indicate stage‐specific alterations in the composition of Ago‐2 associated small RNAs following PPS exposure, with notable shifts in tsRNA length and abundance across epileptogenesis. While these patterns are consistent with the remodeling of Ago2‐bound tsRNA populations, they may also reflect broader changes in Ago2 dynamics or altered competition with other small RNAs such as miRNAs, which are known to fluctuate during disease progression. Thus, the observed changes likely represent a combination of both tsRNA‐specific regulation and context‐dependent redistribution with the Ago2 complex. These observations set the stage for identifying functional targets and pathways potentially influenced by these small RNA regulators in subsequent analyses.

### 3.2 Day of First Seizure (DOFS) Marks a Molecular Convergence Point of Regulatory Disruption Across Small RNA, Transcriptome, and Proteome Layers

To understand how PPS‐induced alterations in tsRNAs might influence molecular networks, we systematically examined regulatory changes at the transcriptome and proteome levels across multiple disease stages. To determine whether the tsRNA dynamics we observed correspond to functional dysregulation of mRNA and protein expression, we leveraged hippocampal RNA‐Seq and proteomics datasets matched to our small RNA‐Seq samples. While earlier studies centered on the chronic epilepsy stage, we shifted our primary focus to DOFS, which is a biologically critical inflection point marking the onset of spontaneous seizures and a potential driver of irreversible network dysfunction.

**FIGURE 4:**
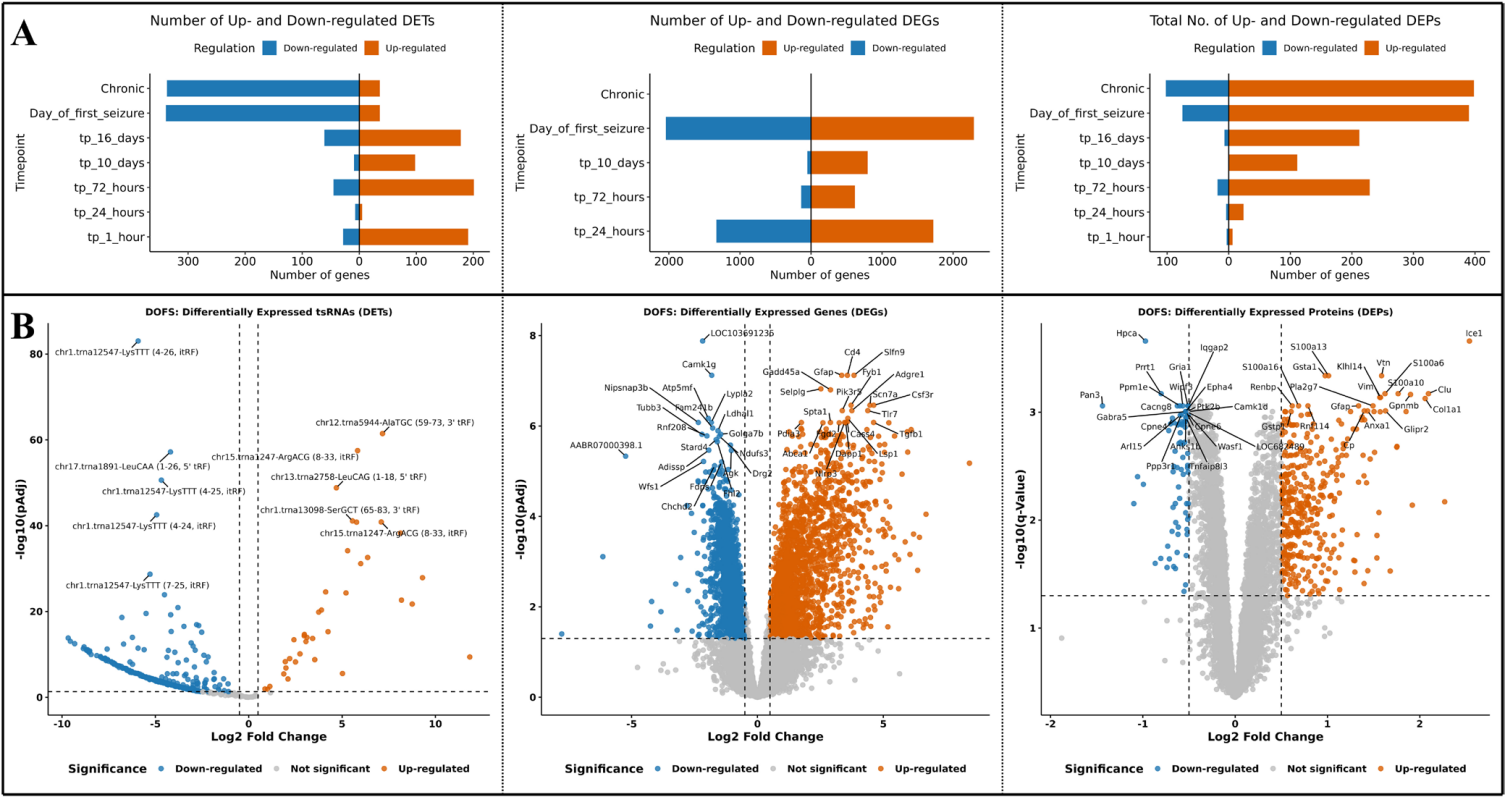
Multi‐omics shifts over time highlight DOFS as a point of coordinated transcriptomic, proteomic, and small RNA dysregulation. (A) Bar plot showing the number of significantly upregulated (red) and downregulated (blue) transfer RNA‐derived small RNA (tsRNAs) (left), genes (center), and proteins (right) across perforant pathway stimulation (PPS)‐exposed timepoints (*n* = 3 rats per timepoint) relative to shared controls (*n* = 3 rats). tsRNAs show upregulation during early stages and sharp downregulation at the day of first seizure (DOFS) and chronic stages. Transcriptomic dysregulation peaks at 24 h and DOFS, while proteomic changes intensity at DOFS and chronic stages. Bars represent count of different expressed features passing adjusted *p*‐value (Benjamini–Hochberg FDR) < 0.05 and |log2FC| > 0.5 threshold. (B) (Left) Volcano plot showing tsRNAs significantly upregulated (red) or downregulated (blue) in PPS‐exposed (*n* = 3 rats per timepoint) versus control samples (*n* = 3 rats) at DOFS. A marked downregulation of functionally active small RNAs is observed at this stage. (Center) Volcano plot showing differentially expressed genes (DEGs) in PPS‐exposed versus control sample at DOFS. Both up‐ and down‐regulated genes are present, indicating widespread transcriptional reprogramming during seizure onset. (Right) Volcano plot showing differentially expressed proteins (DEPs) in PPS‐exposed versus control samples at DOFS. Protein‐level changes intensify at DOFS relative to earlier timepoints.

Distinct temporal patterns of dysregulation at the levels of tsRNAs, mRNAs, and proteins were discovered using a cross‐omics comparison (Figure 4A). At the tsRNAs level (left), a wavelike trend was observed where tsRNAs were globally upregulated during early and intermediate stages following PPS induction. This was followed by a sharp decline in expression at DOFS and chronic stages. At the RNA‐Seq level (middle), broad upregulation was evident during intermediate phases, whereas both the 24 h and DOFS showed substantial dysregulation in both directions. Notably, at the chronic timepoint, no genes passed the significance threshold. This suggests stabilization or loss of responsiveness at the transcript level. Changes at the protein level (right), in contrast, were moderate during early stages. However, at DOFS and chronic phases, the protein level changes became more pronounced, which points to a delayed but progressive impact on protein homeostasis.

Next, we focused on the DOFS timepoint, as that is where all three omics layers exhibit significant changes. Volcano plots of tsRNAs, mRNAs, and proteins at DOFS (in the same order from left to right) showed widespread dysregulation (Figure 4B). This is the stage where the number of up‐ and down‐regulated features across each layer significantly increase. These observations reinforce that DOFS is a molecular convergence point, which is a timepoint or stage where early compensatory mechanisms may give way to pathogenic cascades driving epilepsy onset.

Together, these findings underscore DOFS as a critical regulatory turning point, where coordinated shifts in small RNAs, genes, and proteins may collectively rewire neuronal or glial responses to PPS exposure. These disruptions, therefore, may provide the platform for identifying disease‐associated targets, regulatory modules, and molecular drivers in subsequent integrative analyses.

### 3.3 WGCNA Identifies Distinct Transcriptional Programs at 24 h and DOFS, With tsRNAs Co‐Regulated in the Acute Phase

To uncover coordinated shifts in gene and tsRNA expression during epileptogenesis, we applied WGCNA to variance‐stabilized counts from both RNA‐Seq and small RNA‐Seq datasets. This integrative strategy enabled unbiased clustering of transcripts into modules based on shared expression patterns across PPS‐exposed and control hippocampal samples. To do this, we focused on five timepoints where both datasets were available, that is, 24 h, 72 h, 10 days, DOFS, and chronic epilepsy.

**FIGURE 5:**
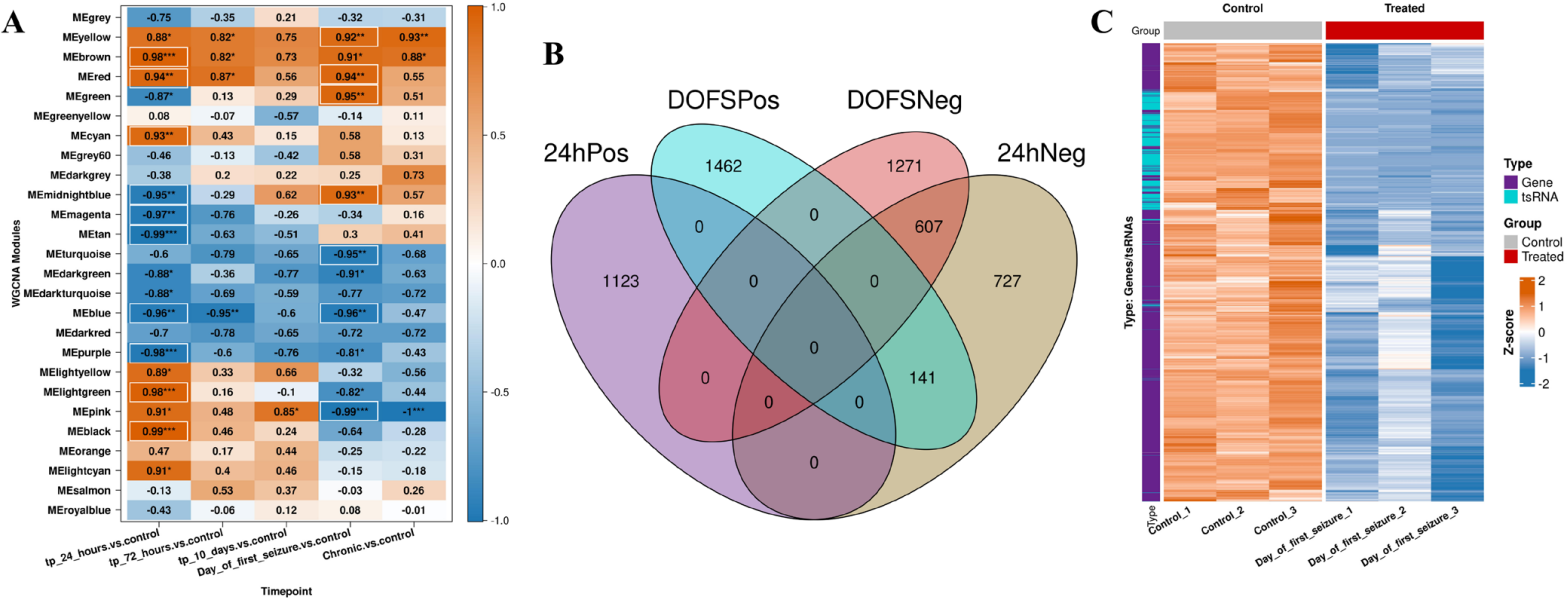
Weighted gene co‐expression network analysis identifies timepoint‐specific modules and highlights DOFS‐associated clusters of co‐regulated tsRNAs and genes. (A) Weighted gene co‐expression network analysis (WGCNA) identified modules of co‐regulated genes and transfer RNA‐derived small RNA (tsRNAs) across five matched timepoints (*n* = 3 rats per group). The strength of association between each module and timepoint is referred to as the module‐trait correlation, which reflects how strongly the average expression of each module relates to the disease condition at a given stage. Heatmap shows correlation between module eigengenes (its first principal component) and timepoints (Perforant pathway stimulation (PPS)‐exposed vs. control (*n* = 3 rats)). Colors indicate strength and direction of correlation (red = positive; blue = negative), with asterisk denoting statistical significance. 24 h and day of first seizure (DOFS) modules showed strong correlations. (B) Venn diagram showing unique and overlapping high‐connectivity genes from combined positively and negatively correlated WGCNA modules at 24 h and DOFS. tsRNAs were found only within the negatively correlated DOFS module (the “pink module”, labeled MEpink in WGCNA output), highlighting their specificity to the acute seizure phase. (C) Expression heatmap of genes and tsRNAs clustered together in the negatively correlated DOFS modules showing variance‐stabilized counts for samples in DOFS and control groups. tsRNAs (annotated in cyan) are co‐downregulated with a subset of mRNAs (annotated in purple) specifically in PPS‐exposed DOFS samples.

Several co‐regulated modules were identified across timepoints using WGCNA. Notably, the strength of association between each module and experimental conditions, referred to as the module‐trait correlation, was particularly high at 24 h and DOFS (Figure 5A). In simple terms, these module‐trait correlations show how strongly a cluster of co‐expressed genes and tsRNAs is associated with a specific stage of epileptogenesis. At 24 h and DOFS, both positively correlated (upregulated in PPS‐exposed samples) and negatively correlated (downregulated in PPS‐exposed samples) modules were identified, suggesting the presence of distinct transcriptional programs that are either activated or suppressed at these key stages of disease progression.

To further characterize these modules, we identified highly connected “hub” genes and tsRNA, those showing strong correlation with their respective module's overall expression profile (|kME| > 0.8). This highlights the most central and potentially functionally important members of each co‐expression cluster. A Venn diagram (Figure 5B) illustrates how these high‐connectivity genes overlap or differ between positively and negatively correlated modules at 24 h and DOFS.

Interestingly, tsRNAs appeared exclusively within one of the most significantly downregulated modules at DOFS (the “pink module”, labeled MEpink in WGCNA output), indicating that these tsRNAs are part of a transcriptional program that is collectively reduced during the acute seizure phase (Figure 5C). No tsRNAs were detected in modules upregulated at DOFS or at the earlier 24‐h stage. This pattern suggests that tsRNA‐associated regulation may be particularly affected at seizure onset, possibly reflecting a transient loss or remodeling of post‐transcriptional control mechanisms.

Although tsRNAs have been proposed to act as miRNA‐like repressors, their co‐downregulation alongside genes in the DOFS‐negatively correlated modules challenges a simplistic interpretation. It is possible that tsRNAs serve indirect or stress‐related regulatory functions such as tuning translation, buffering stress granule dynamics, or modulating upstream transcriptional regulators. Their disappearance from active modules may reflect a breakdown in post‐transcriptional control or a switch in their functional role during acute epileptogenesis.

To further understand the biology represented in these modules, we performed gene set enrichment analysis on the unique high‐kME genes from the positively and negatively correlated modules at 24 h and DOFS.

### 3.4 Distinct WGCNA Modules at DOFS and 24 h Reveal Diverging Molecular Programs Anchoring tsRNAs to Suppressed Metabolic and Synaptic Processes

To functionally characterize the distinct transcriptional programs uncovered by WGCNA, we performed gene ontology biological process (GO BP) and KEGG pathway enrichment analysis on high‐confidence genes (kME > 0.8) from each of the four major co‐expressed modules at 24 h and DOFS (positively and negatively correlated modules separately). This analysis offers insight into the biological roles of genes in each cluster and lays the foundation for linking tsRNAs to functional outcomes.

**FIGURE 6:**
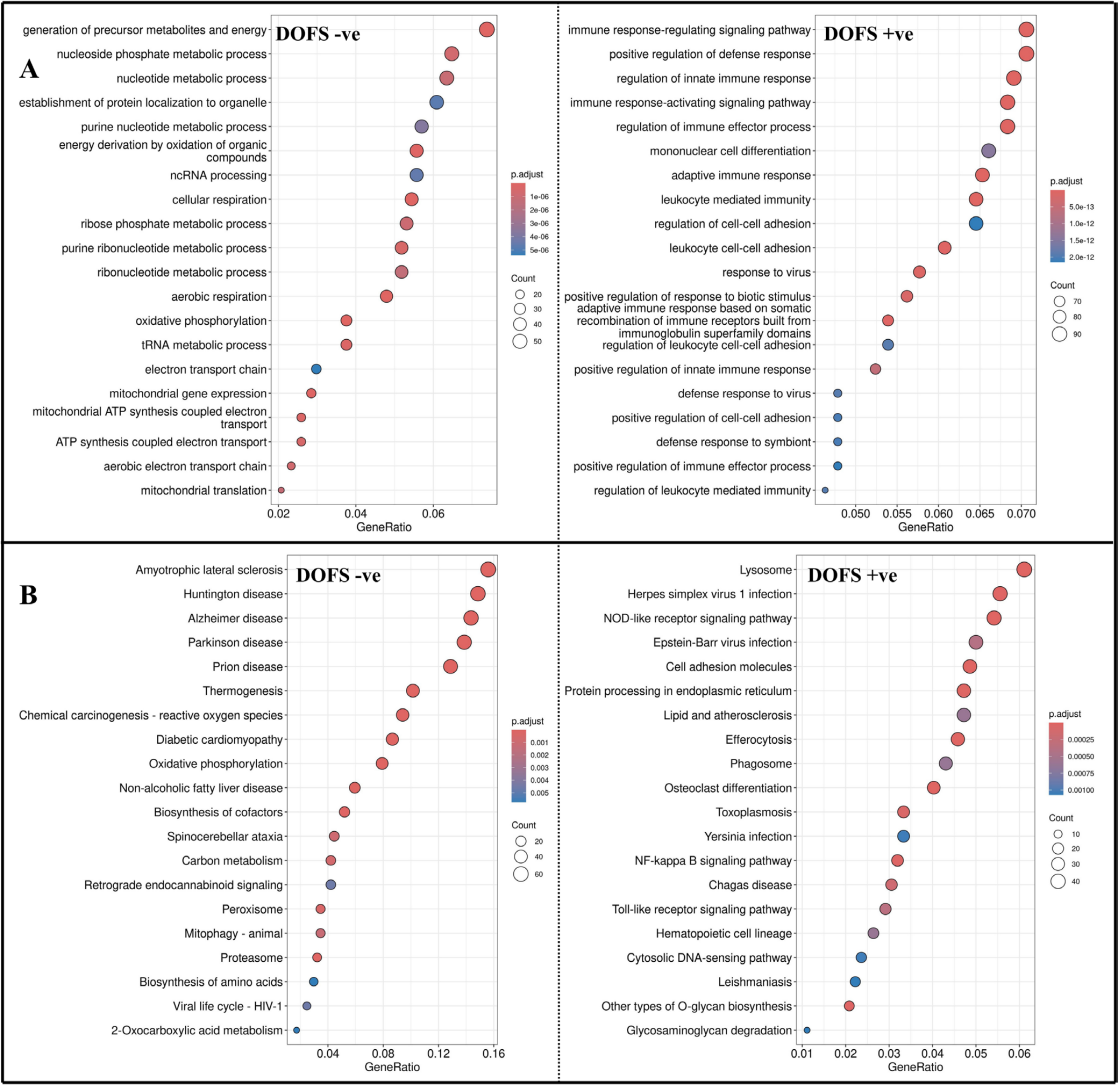
Functional annotation of DOFS‐associated gene modules reveals divergent Immune activation and metabolic suppression. (A) (Left): Gene ontology (GO) biological processes enriched in the negatively correlated the day of first seizure (DOFS) modules which contained all transfer RNA‐derived small RNA (tsRNA). Terms include mitochondrial respiration, oxidative phosphorylation, and nucleotide metabolism, which are pathways central to neuronal energy and synaptic maintenance. Genes associated with these pathways were significantly downregulated in perforant pathway stimulation (PPS)‐exposed samples (*n* = 3 rats per timepoint) at seizure onset. (B) (left): Kyoto Encyclopedia of Genes and Genomes (KEGG) pathways in the DOFS‐negatively correlated modules reflect metabolic suppression and neurodegeneration including Alzheimer's, Parkinson's, and Huntington's disease pathways. The coordinated downregulation suggests energy collapse and cellular stress during seizure onset. (A) (right): GO biological processes enriched in the DOFS‐positively correlated modules include innate immune response, leukocyte activation, and cytokine signaling, which are consistent with neuroinflammatory signaling during acute seizure onset. (B) (right): KEGG terms show activation of immune pathways including Toll‐like receptor and NF‐kappa B signaling suggesting early activation of neuroimmune responses coinciding with seizure onset.

The negatively correlated modules at DOFS, one of which (the “pink module”, labeled MEpink in WGCNA output) notably contained all tsRNAs, revealed strong enrichment for mitochondrial respiration, oxidative phosphorylation, and nucleotide metabolism. GO terms such as aerobic respiration, ATP synthesis, mitochondrial translation, and tRNA metabolic process (Figure 6A, left) reflect a broad suppression of neuronal energy homeostasis. KEGG enrichment reinforced this interpretation with terms like Parkinson disease, Huntington disease, Alzheimer's disease, oxidative phosphorylation, and mitophagy (Figure 6B, left), which are canonical hallmarks of mitochondrial stress and neurodegenerative vulnerability. The co‐downregulation of tsRNAs with these genes suggests a potential breakdown of pathways supporting neuronal energy metabolism at seizure onset.

The positively correlated modules at DOFS were enriched for immune‐related signaling and leukocyte activation, for example, positive regulation of innate immune response, B cell activation, cytokine receptor interaction, and NOD‐like receptor signaling (Figure 6A, right). KEGG terms such as Toll‐like receptor pathway, NF‐kappa B signaling, and viral response pathways highlight neuroinflammatory engagement during DOFS (Figure 6B, right). These patterns are consistent with an acute inflammatory cascade that accompanies seizure onset and may interact with neurodegenerative processes identified above.

Notably, this strong enrichment for immune and cytokine‐related processes, including pathways connected to interleukin signaling shown in the DOFS positively correlated mRNA module, is previously reported by Siebenbrodt et al. (2022), where they observed marked hippocampal IL‐1β elevation peaking during epileptogenesis, as well as sustained IL‐10 levels in the same PPS model. This overlap suggests that the transcriptional immune signature that we see around DOFS reflects an active inflammatory state that is consistent with independent experimental observations.

In contrast, 24 h timepoint modules revealed different processes. The 24 h negatively correlated modules were enriched for synaptic structure and signaling pathways including synapse assembly, regulation of membrane potential, glutamate receptor signaling, and learning or memory (Figure S1A, left). These terms suggest early disruption in neuronal connectivity and excitability, even before the first seizure. KEGG analysis supported this by showing enrichment for glutamatergic, GABergic, and cholinergic synapses, long‐term potentiation, and calcium signaling (Figure S1B, left).

Lastly, the 24 h positively correlated modules showed enrichment for cell cycle‐related processes such as chromosome segregation, DNA replication, mitotic transition, and cell division (Figure S1A, right), alongside KEGG terms for cell cycle, p53 signaling, apoptosis, and NF‐kappa B activation (Figure S1B, right). These findings reflect transcriptional priming, potentially marking glial activation, proliferative stress, or apoptotic vulnerability at early stages of epileptogenesis.

Taken together, these enrichments point toward a temporally bifurcated pathology: early excitatory and proliferative signaling at 24 h, and metabolic suppression plus immune activation at DOFS. The fact that tsRNAs co‐cluster exclusively in the downregulated, mitochondrial‐enriched DOFS modules suggests their potential role as suppressive regulators of critical neuronal survival pathways and frames DOFS as a molecular inflection point where homeostatic programs falter.

To test whether tsRNAs could actively mediate such repression, we next examined tsRNA‐mRNA targeting relationships using sequence‐based hybridization models.

### 3.5 tsRNAs at DOFS May Act as Suppressors of Neuronal Metabolic Pathways via Direct Targeting of Key Transcripts

Having established that tsRNAs co‐clustered with genes downregulated in mitochondrial and metabolic pathways at DOFS, we next sought to determine whether tsRNAs may influence these gene programs via sequence‐specific interactions with transcripts. To achieve this, we used RNAhybrid to perform target prediction, focusing on differentially expressed tsRNAs identified at DOFS and testing their potential binding to differentially expressed genes and proteins from RNA‐Seq and proteomics datasets, respectively. To maximize biological relevance, we considered only genes that were differentially expressed in at least one omics layer (transcript or protein) and unified them into a single candidate pool.

We extracted 5′ UTR, 3′ UTR, and CDS sequences of these genes using Ensembl BioMart and screened all sequences for potential hybridization with tsRNAs using RNAhybrid. To reduce false positives, we retained only hits with minimum free energy (MFE) ≤ −20 kcal/mol and *p*‐value < 0.05, indicative of highly stable interactions.

**FIGURE 7:**
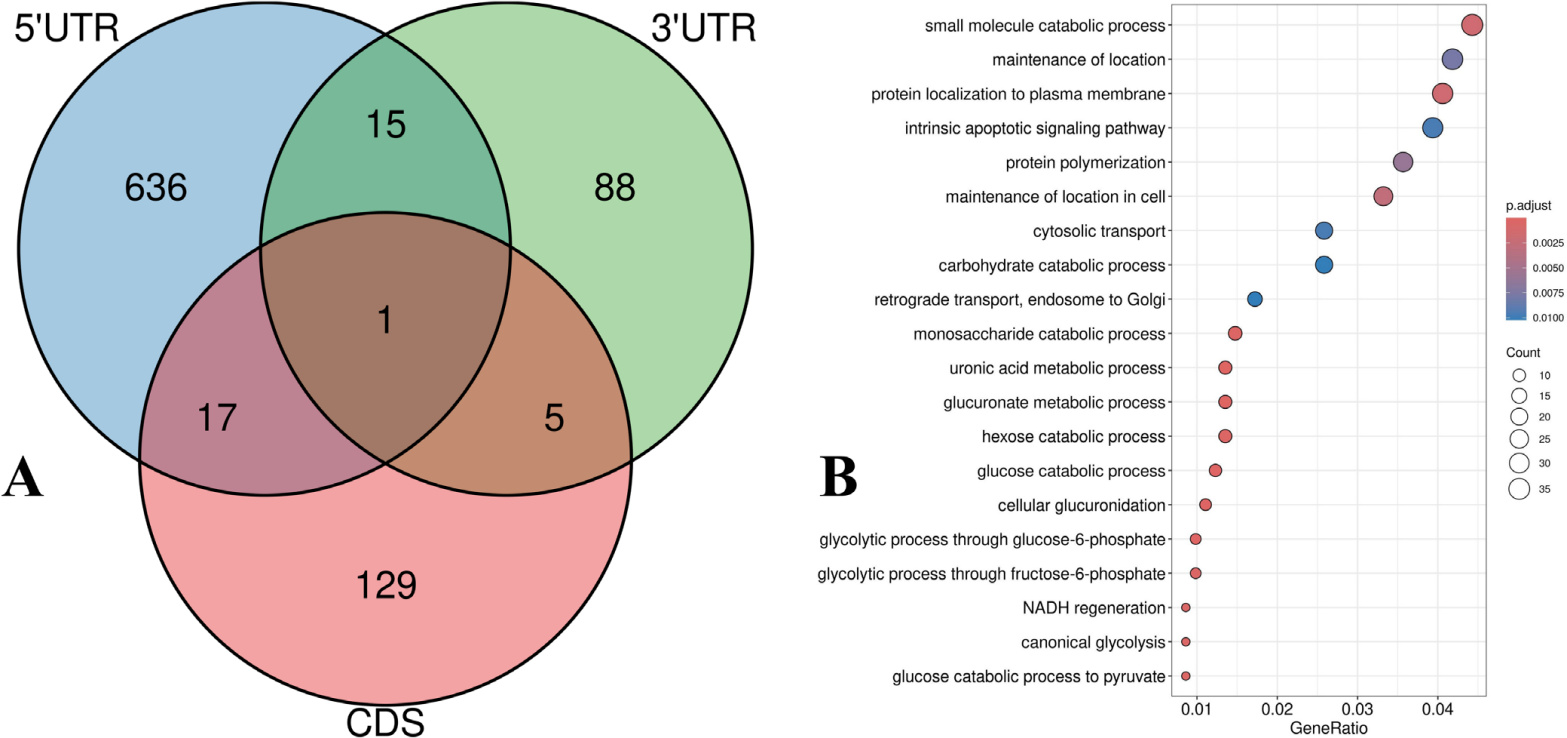
Predicted tsRNA binding sites and GO enrichment highlight metabolic and transport pathways. (A) Venn Diagram illustrates transfer RNA‐derived small RNA (tsRNA) targets predicted across 5′ untranslated region (5′ UTR), 3′ untranslated region (3′ UTR), and coding sequence (CDS). RNA hybrid was used to predict target regions of differentially expressed tsRNAs at the day of first seizure (DOFS). The majority of predicted tsRNA binding was localized to 5′ UTRs, followed by CDS and 3′ UTRs. Only interaction with minimum free energy (MFE) ≤ −20 kcal/mol and *p* < 0.05 were considered. (B) Gene ontology (GO) biological processes for predicted tsRNA‐targeted genes were enriched for energy metabolism (glycolysis, glucose catabolism), intracellular transport (endosomal transport, vesicle localization), cytoskeletal organization (actin polymerization), and regulated exocytosis. These functions are essential for neuronal maintenance and align with previous WGCNA results showing suppression of similar pathways.

The results showed that tsRNAs were predicted to bind across multiple regions of mRNAs, with the highest number of predicted interactions in 5′ UTRs, followed by CDS and 3′ UTR. Interestingly, while most targets were region‐specific, a small subset showed multi‐region binding (Figure 7A). These findings are consistent with emerging evidence that tsRNAs can bind beyond traditional miRNA‐like 3′ UTR sites, including in 5′ UTR and CDS regions, potentially modulating transcript stability, ribosome occupancy, or translation elongation (Gu et al. 2022; Kim et al. 2019).

To explore the biological roles of these predicted target genes, we performed GO enrichment analysis on the union of all region‐specific predicted targets. The analysis revealed a strong overrepresentation of glycolysis, glucose catabolism, and metabolic transport, as well as vesicle trafficking, cytoskeletal dynamics, and protein localization to synapses and membranes (Figure 7B). This aligns closely with the WGCNA enrichment of the DOFS‐negative modules, where tsRNAs were co‐downregulated with mitochondrial and metabolic genes, reinforcing the hypothesis that tsRNAs may act as regulators or facilitators of energy metabolism and vesicular dynamics in neurons.

Together, these findings link tsRNA co‐expression and co‐targeting patterns, suggesting that tsRNAs downregulated at DOFS could potentially bind and influence genes involved in neuronal energetics, exocytosis, and trafficking, which are all crucial for maintaining synaptic function during epileptogenesis.

To further evaluate whether tsRNAs and their predicted mRNA targets exhibit coordinated expression in vivo, we performed a correlation‐based validation analysis at the DOFS timepoint (see Data S1 (Methods, Results) and Figure S2). Predicted tsRNA‐mRNA pairs displayed region‐specific correlation patterns across 3′ UTR, CDS, and 5′ UTR binding regions (Figure S2A,B). 3′ UTR and CDS pairs showed predominantly negative correlations (median *p* ≈ −0.6), consistent with repressive post‐transcriptional regulation, while 5′ UTR pairs exhibited a more balanced, bimodal distribution of positive and negative associations (median *p* ≈ +0.5). These distributions significantly differed from random tsRNA‐gene pairings (Kolmogorov–Smirnov test *p* < 10^−10^), supporting that tsRNA‐target relationships reflect structured expression patterns rather than random noise.

We next asked whether this set of putative tsRNA targets is linked to neurological disorders by examining known disease associations.

### 3.6 Predicted Targeted Genes Are Enriched in Neurological Disorders, Including Epilepsy

To evaluate the clinical relevance of genes predicted to be targeted by tsRNAs at DOFS, we performed gene‐disease association analysis using DisGeNET, a curated database linking genes to disease phenotypes. Since DisGeNET is human‐centric, we first mapped rat genes to their human orthologs, retaining only those with confirmed matches in DisGeNET's curated dataset.

**FIGURE 8:**
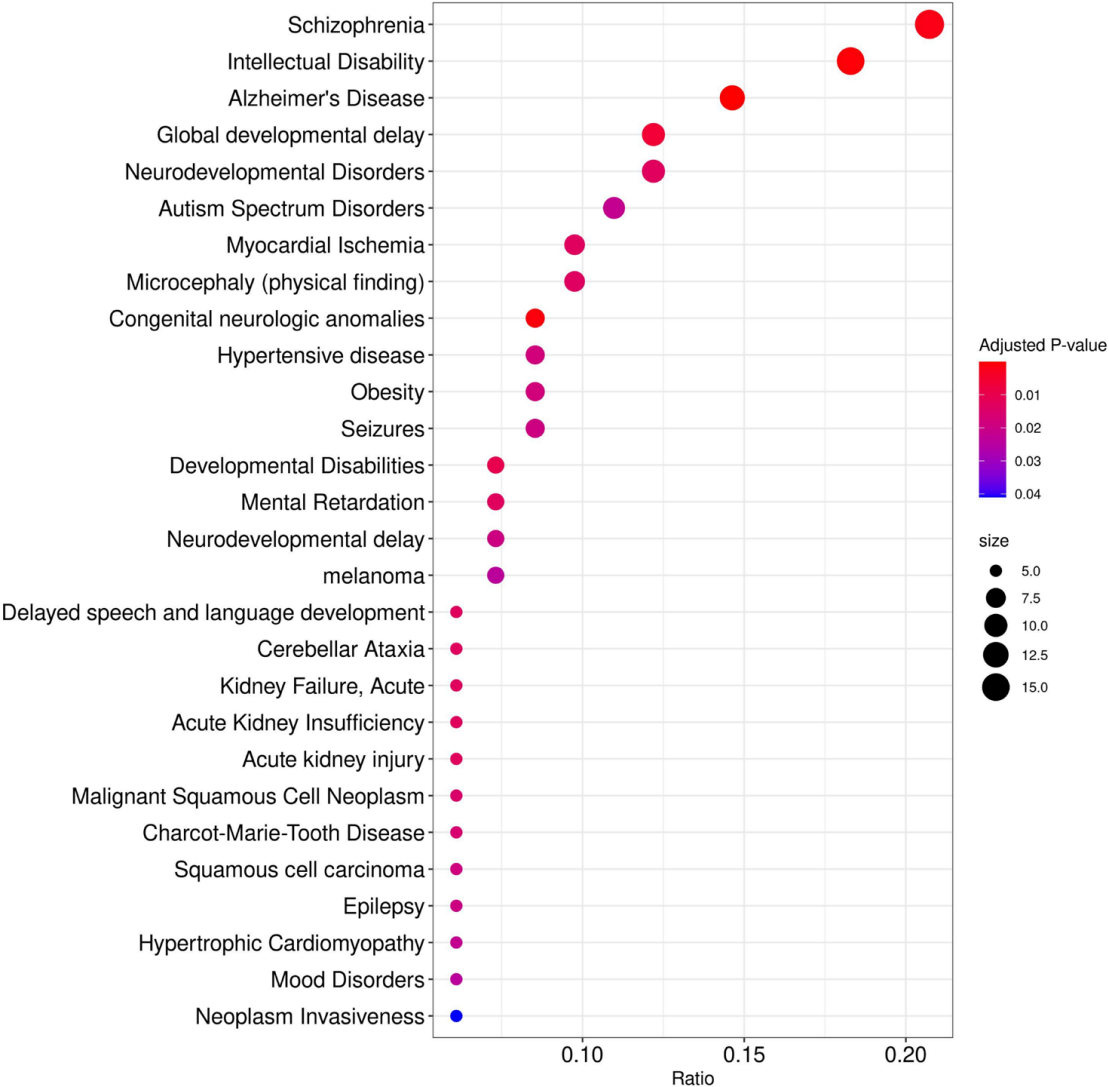
Gene‐disease enrichment analysis of tsRNA predicted targets using DisGeNET. DisGeNET‐based gene‐disease association analysis of transfer RNA‐derived small RNA (tsRNA)‐predicted target genes (derived from perforant pathway stimulation (PPS)‐exposed day of first seizure (DOFS) samples, *n* = 3 rats). Genes were mapped to human orthologs prior to enrichment testing. Predicted targets showed associations with neurological and neurodevelopmental disorders, including epilepsy.

The analysis revealed that predicted tsRNA target genes are significantly enriched in a range of neurodevelopmental and neurodegenerative disorders (Figure 8). These include epilepsy‐related conditions such as seizures, epilepsy, and autism spectrum disorders; neurodegenerative diseases such as Alzheimer's disease and cerebellar ataxia; and neurodevelopmental impairments such as intellectual disability, delayed speech and language development, microcephaly, and global developmental delay. Additional associations with mood disorders, schizophrenia, and Charcot–Marie–Tooth disease further support the nervous system‐specific impact of tsRNA regulation.

Notably, several high‐confidence epilepsy genes were among the predicted targets, including STXBP1, associated with epileptic encephalopathies; SYNGAP1, a known gene for developmental delay and seizures; WDR45, implicated in neurodegeneration with brain iron accumulation; EEF1A2 and ACHE, linked to epilepsy, synaptic transmission, and muscular disorders.

This enrichment suggests that tsRNAs differentially expressed in DOFS are not merely passive biomarkers but may directly contribute to the transcriptomic and proteomic alterations underlying epilepsy‐related pathology.

Together, our analyses reveal that tsRNAs exhibit stage‐specific expression patterns, with a marked collapse at DOFS. At this inflection point, tsRNAs co‐cluster with genes involved in neuronal energy metabolism and synaptic maintenance, show direct sequence‐based targeting of those same pathways, and are enriched in neurological disease relevance, including epilepsy.

## 4 Discussion

Utilizing an established rat model of TLE induced by PPS, this study represents the first systematic, cross‐omics investigation of Ago2‐bound tsRNA derived small RNAs (tsRNAs) during epileptogenesis. By integrating the dynamics of tsRNAs with transcriptome and proteome datasets at defined stages of disease progression, we found the DOFS timepoint as a molecular inflection point where a convergence of regulatory collapse and functional remodeling occurs. We demonstrate that tsRNAs, particularly those bound to Ago2 and therefore functionally active, exhibit time‐dependent regulation, with widespread downregulation at DOFS and chronic stages. WGCNA discovered tsRNAs co‐expressed with genes involved in energy metabolism and synaptic homeostasis. Subsequently, target prediction, enrichment analysis, and gene‐disease enrichment linked those genes to pathways involved in mitochondrial function, vesicular transport, and pathways previously associated with neurodevelopmental disorders and epilepsy. Together, the findings point to a possible contribution of tsRNAs to the molecular changes that accompany epileptogenesis and suggest new avenues for exploring ncRNA‐based regulation in epilepsy.

While miRNAs remain the most studied small ncRNAs in epilepsy and other neurological disorders, tsRNAs are becoming increasingly important in brain diseases (Winek and Soreq 2025). Our analyses indicate that Ago2‐bound tsRNAs, particularly 5′ tiRNAs and 3′ tRFs, are dynamically modulated across epileptogenesis and may play distinct roles at different stages. Early to intermediate stages showed global upregulation of tsRNAs, consistent with stress‐induced small RNA responses described in other systems (Emara et al. 2010). In contrast, DOFS and chronic stages displayed a marked reduction in short tsRNA abundance and accumulation of longer 5′ tiRNAs. This remodeling suggests a potential shift in post‐transcriptional control as neuronal networks transition to a seizure‐generating state. Such stage‐specific shifts may reflect loss of small RNA‐mediated fine‐tuning and increased transcriptional noise associated with disease progression.

Our cross‐omics analysis highlights DOFS as a unique convergence zone, where dysregulation in genes intensifies and protein level alterations become most pronounced. DOFS is an important timepoint, representing a short temporal window when the hippocampal and connected networks transition to a state capable of generating spontaneous recurrent seizures, that is, epilepsy. That cells within the hippocampus engage unique programs of molecular control suggests either a final step in the process of epileptogenesis or perhaps attempts at resilience, that represent ultimately failed attempts to oppose synchronous network development. Notably, other non‐coding RNA species also seem to undergo marked changes during the DOFS, including circular RNAs (Gomes‐Duarte et al. 2021). We recognize as well that the DOFS sampling may reflect molecular changes uniquely caused by a first spontaneous seizure that do not persist in the chronic state. This lays open the possibility of both unique biomarkers of the establishment of the early epileptic state as well as a point where perhaps therapeutic intervention may still be possible to avoid chronic epilepsy.

Our WGCNA results reinforce this interpretation. DOFS modules showed the strongest co‐regulatory signatures, including a negatively correlated cluster of genes enriched in mitochondrial respiration, protein transport, and neuronal structural integrity. tsRNAs were enriched in this module, suggesting that they may participate in the coordinated regulation of these processes. While traditional assumptions frame tsRNAs as suppressors, some may also stabilize or fine‐tune gene expression (Chu et al. 2022; Kim et al. 2017). Their co‐clustering with downregulated targets suggests a broader regulatory capacity, perhaps influencing translation, localization, or RNA turnover beyond classical silencing. The immune‐related module identified at DOFS also aligns with evidence of cytokine activation in previous studies. Specifically, the work by Siebenbrodt et al. (2022) reported strong IL‐1β elevation and sustained IL‐10 increases around this disease stage using hippocampal microdialysis in a similar PPS model. The harmony between transcriptional immune signatures in our study and in vivo cytokine levels supports the notion that seizure onset coincides with an inflammatory milieu. Because certain Ago2‐bound tsRNAs identified in our data may target mRNAs involved in these immune pathways, they could be contributing to or responding to this neuroinflammatory shift.

Target prediction analyses suggested that tsRNAs at DOFS may interact with transcripts involved in glycolysis, vesicle trafficking, and cytoskeletal organization, which are processes fundamental to synaptic function and neuronal plasticity (Gordon‐Weeks and Fournier 2014; McDonald et al. 2018; Van Liefferinge et al. 2013). These pathways are also known to be disrupted in epilepsy and related disorders. The potential for tsRNAs to engage such networks is intriguing and warrants further biochemical validation. Gene‐disease enrichment analysis revealed significant associations between predicted tsRNA targets and neurological conditions including epilepsy, autism spectrum disorder, and neurodegeneration. STXBP1, SYNGAP1, and WDR45, which are canonical epilepsy genes, were among the predicted targets, suggesting that tsRNAs may contribute to the broader molecular landscape underlying disease susceptibility (Zhang et al. 2024). Together, these findings add new layers to the understanding of sncRNAs involvement in neurological disorders and suggest that tsRNAs might participate in molecular processes underlying epileptogenesis.

This study is computational in nature and therefore limited by the absence of experimental validation of tsRNA‐mRNA interactions. While Ago2 immunoprecipitation enriches for functionally active RNAs, not all Ago2‐bound tsRNAs necessarily exert regulatory effects and may act through Ago‐independent mechanisms such as stress granule formation or apoptotic signaling (Emara et al. 2010; Gebetsberger et al. 2017; Kumar et al. 2014; Maute et al. 2013; Saikia et al. 2014). In addition, it is important to note that the observed shifts in Ago2‐bound tsRNA composition during epileptogenesis may not solely reflect changes in tsRNA biogenesis or degradation. Alterations in Ago2 expression, post‐translational modification, or competitive leading with other small RNAs such as miRNAs could also influence the observed distribution of Ago2‐associated small RNAs. Thus, the changes we report likely represent a combined effect of tsRNA‐specific dynamics and broader remodeling of the Ago2 complex during disease progression. In addition, target predictions were based on in silico thermodynamic models and require biochemical confirmation. To partly address this limitation, we performed a computational correlation‐based validation (see Data S1 (Methods, Results) and Figure S2) that demonstrated region‐specific tsRNA‐mRNA expression relationships distinct from random expectation. Nevertheless, these correlations are associative and not proof of direct regulation. Future functional assays (e.g., luciferase reporter or knockdown studies) will be required to establish causality. Finally, cross‐species mapping between rat and human orthologs may introduce bias in disease enrichment analyses.

In summary, this study provides a system‐level view of Ago2‐bound tsRNAs in an established model of TLE, showing that tsRNAs undergo pronounced temporal shifts, particularly at the onset of spontaneous seizure. The integration of tsRNA, transcriptomic, and proteomic data identifies DOFS as a molecular transition point characterized by widespread regulatory remodeling. While causal mechanisms remain to be tested experimentally, these findings underscore the potential for tsRNAs to participate in the molecular reprogramming that accompanies epileptogenesis and highlight promising directions for future functional and therapeutic exploration.

## Author Contributions

**Saad Zaheer:** conceptualization, software, formal analysis, writing – original draft, writing – review and editing, methodology. **Sharada Baindoor:** software, resources, writing – review and editing. **Niamh M. C. Connolly:** software, resources, writing – review and editing. **Kai Siebenbrodt:** writing – review and editing, resources. **Sebastian Bauer:** investigation, writing – review and editing. **Felix Rosenow:** investigation, funding acquisition, writing – review and editing. **Jens S. Andersen:** investigation, funding acquisition. **Morten T. Venø:** investigation, resources, writing – review and editing. **Jørgen Kjems:** investigation, funding acquisition, writing – review and editing. **David C. Henshall:** conceptualization, funding acquisition, writing – review and editing. **Jochen H. M. Prehn:** conceptualization, funding acquisition, writing – original draft, writing – review and editing, supervision, methodology.

## Funding

This work was supported by the Taighde Éireann—Research Ireland Centre for Research Training in Genomics Data Science under Grant 18/CRT/6214. J.H.M.P., D.C.H., J.K., and M.T.V. were supported by Horizon 2020 ‘PRIME—A Personalised Living Cell Synthetic Computing Circuit for Sensing and Treating Neurodegenerative Disorders’ (H2020 FET—GA 964712). J.H.M.P. received support from Horizon 2020 ‘InterTau—Integrative structural biology of pathological tau protein, an appealing therapeutic target for Alzheimer's disease modifying drugs (H2020 MSCA RISE GA 873127)’. J.H.M.P. and D.C.H. were supported by Taighde Éireann—Research Ireland, under Grant number 21/RC/10294_P2 at FutureNeuro Research Ireland Centre for Translational Brain Science. This work was also supported by the European Union's “Seventh Framework” Programme (FP7) under Grant Agreement 602 130 (EpimiRNA).

## Conflicts of Interest

The authors declare no conflicts of interest.

## Supporting information


**Data S1:** jnc70317‐sup‐0001‐FigureS1‐S2.docx.

## Data Availability

The sequencing datasets are available on Gene Expression Omnibus GSE137473, and the proteomics data are available on Proteomics Identifications Database (PRIDE) PXD019098. All differential expression analysis results, and relevant metadata files are reported in the Supporting Information files.
