# Supplementary material for: Temporal Dynamics of tsRNA Regulation Mark an Abrupt Transition After Epileptogenesis
Source: J Neurochem. 2025 Dec 15;169(12):e70317. doi: 10.1111/jnc.70317 (PMC12703685; doi:10.1111/jnc.70317)
Supplement: Supplementary file 1 — Data S1: jnc70317‐sup‐0001‐FigureS1‐S2.docx. [file JNC-169-0-s001.docx]

**Supplementary Information for:**

**Temporal Dynamics of tsRNA Regulation Mark an Abrupt Transition After Epileptogenesis**

Saad Zaheer^1,2^, Sharada Baindoor^1,2^, Niamh M. C. Connolly^1,2^, Kai Siebenbrodt^3,4^, Sebastian Bauer^3,4^, Felix Rosenow^3,4^, Jens S. Andersen^5^, Morten T. Venø^7^, Jørgen Kjems^6^, David C. Henshall^1,2^, Jochen H.M. Prehn^1,2*^

^1^Dept. of Physiology & Medical Physics and RCSI Centre for Systems Medicine, RCSI University of Medicine and Health Sciences, Dublin 2, Ireland.

^2^FutureNeuro Research Ireland Centre, RCSI University of Medicine and Health Sciences, Dublin 2, Ireland.

^3^Goethe-University Frankfurt, Epilepsy Center Frankfurt Rhine-Main, Department of Neurology, University Medical Center, Frankfurt, Germany.

^4^Goethe-University Frankfurt, Center for Personalized Translational Epilepsy Research (CePTER), Frankfurt, Germany.

^5^Center for Experimental Bioinformatics, University of Southern Denmark, DK-5230 Odense M, Denmark.

^6^Interdisciplinary Nanoscience Center, Dept. of Molecular Biology and Genetics, Aarhus University, Aarhus, Denmark.

^7^Omiics ApS, Aarhus, Denmark

***Corresponding author:** [**prehn@rcsi.ie**](mailto:prehn@rcsi.ie)

**Supplementary Methods**

**Computational Validation of tsRNA-mRNA Regulatory Associations**

To evaluate whether tsRNAs and their predicted mRNA targets exhibit coordinated expression patterns, we performed a correlation-based validation analysis at the day of first seizure (DOFS) timepoint. Predicted tsRNA-mRNA target pairs were obtained from thermodynamic and sequence complementarity analyses (minimum free energy ≤ -20 kcal/mol, p < 0.05) and categorized according to the predicted binding region (5’UTR, CDS, or 3’UTR).

Raw count matrices for tsRNAs and mRNAs were generated from small RNA-Seq and RNA-Seq datasets obtained from (Venø et al., 2020) and transformed using the variance-stabilizing transformation (VST) implemented in DESeq2 to normalize for sequencing depth and variance heterogeneity. For each predicted tsNRA-gene pair, Spearman’s rank correlation coefficient (p) and its associated p-value were computed across samples. False-discovery rate (FDR) correction was applied to adjust for multiple testing.

To test whether the observed correlations deviated from random expectations, we generated a background distribution by sampling random tsRNA-gene pairs of comparable size to the predicted dataset and computed their correlation coefficients using the same procedure. The resulting distributions were compared using the Kolmogorov-Smirnov (KS) test, which assesses whether two distributions differ significantly in shape or central tendency.

**Supplementary Results**

**Expression Correlations Patterns between tsRNAs and predicted Target Genes**

Across all predicted interactions, correlation values (p) were broadly distributed, spanning both positive and negative ranges (Figure S2). The median correlation coefficients were -0.60 for 3’UTR, and CDS, and +0.49 for 5’UTR predicted targets. These trends indicate that tsRNAs potentially interacting within 3’UTR and CDS regions tend to exhibit inverse expression relationships with their predicted target genes, consistent with canonical post-transcriptional repression, whereas 5’UTR interactions display a more balanced mix of positive and negative associations.

In the density plot (Figure S2A), 3’UTR and CDS distributions were negatively skewed, showing prominent peaks around p ≈ -0.6 to -0.8 and smaller secondary peaks in the positive range. The density plot for 5’UTR exhibited a similar strong bimodal distribution, with high peaks on both the negative and positive sides. Then random control (black line) showed shallow peaks at both positive and negative correlation extremes and a broad middle plateau, reflecting random variation without structured correlation patterns.

The bar plot (Figure S2B) summarizes median p values per region, together with the KS test p-values demonstrating that each distribution significantly differed from random expectation (3’UTR: p = 2.6 x 10^-10^; CDS: p = 2.5 x 10^-14^; 5’UTR: p = 1.6 x 10^-47^). Collectively, these findings suggest that tsRNA expression patterns are not random with respect to their predicted gene targets, and that region-specific interactions may underpin distinct modes of tsRNA-mediated regulation.

**Supplementary Figure S1**

Functional Annotation of 24 hours-Associated Gene Modules


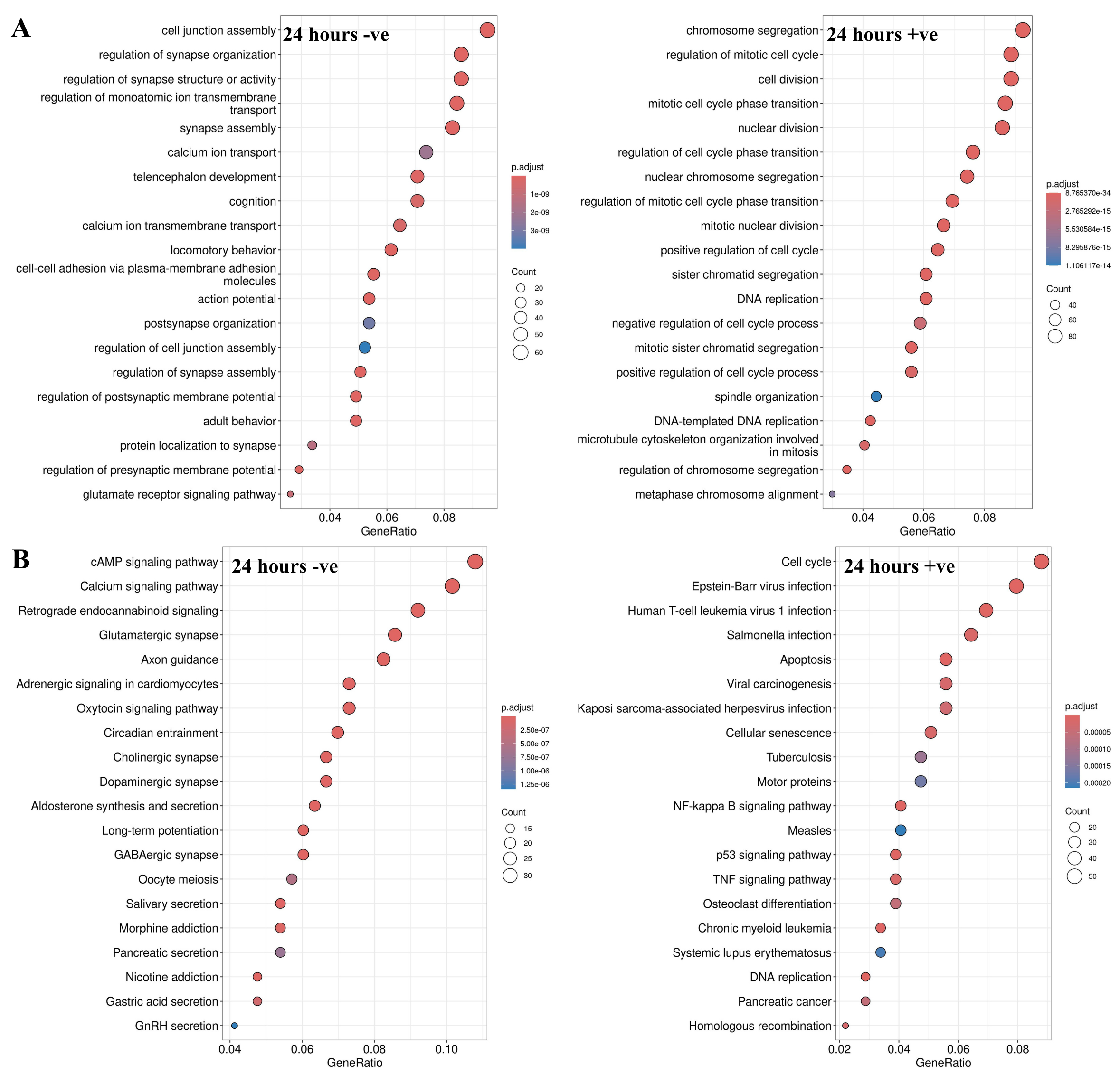


**A (Left):** Gene Ontology (GO) biological processes enriched in the negatively correlated 24 hours modules mostly enriched for synaptic structure and signaling pathways. **B (Left):** Kyoto Encyclopedia of Genes and Genomes (KEGG) pathways in 24 hours-negative modules showing enrichment in glutamatergic, GABergic, and cholinergic synapses. **A (Right):** GO biological processes enriched in the positively correlated 24 hours modules enriched in pathways including chromosome segregation, DNA replication, mitotic transition, and cell division. **B (Right):** KEGG terms enriched in 24 hours positively correlated modules showing enriched pathways such as cell cycle, p53 signaling, apoptosis, and NF-kappa B activation.

**Supplementary Figure S2**

Validation of tsRNA-mRNA expression relationships


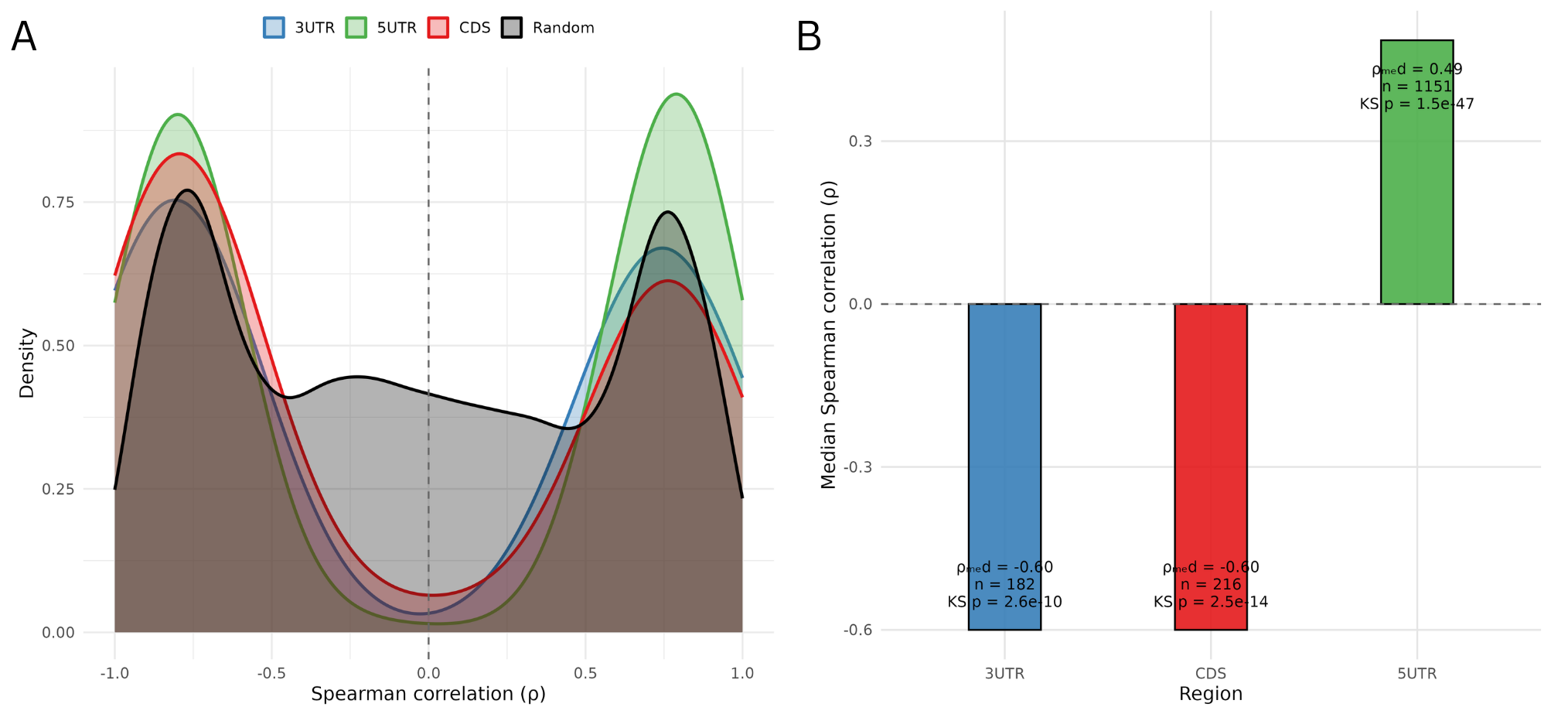


**(A)** Density distribution of Spearman correlation coefficients (p) between tsRNAs and their predicted mRNA targets in 5’UTR (green), CDS (red), and 3’UTR (blue) regions, compared to random tsRNA-gene pairings (black line). 3’UTR and CDS distributions are skewed toward negative correlations, while the 5’UTR shows skewness toward positive correlation. **(B)** Summary of median tsRNA-mRNA correlation coefficients (p) per region. Each bar is annotated with Kolmogorov-Smirnow (KS) p-values comparing the regional correlation distribution to the random background. n denotes the number of predicted tsRNA-gene pairs per region. Negative median p values for 3’UTR and CDS region highlight predominantly inverse expression relationships consistent with potential post-transcriptional repression.

**Supplementary Data Files**

Additional supplementary files including samples metadata and datasets from gene disease association analysis, weighted gene co-expression network analysis (WGCNA), target analysis, and differential expression analysis of RNA-Seq, small RNA-Seq, and proteomics are available at Figshare: <https://doi.org/10.6084/m9.figshare.29919587>.

**References**

Venø, M. T., Reschke, C. R., Morris, G., Connolly, N. M. C., Su, J., Yan, Y., Engel, T., Jimenez-Mateos, E. M., Harder, L. M., Pultz, D., Haunsberger, S. J., Pal, A., Heller, J. P., Campbell, A., Langa, E., Brennan, G. P., Conboy, K., Richardson, A., Norwood, B. A., … Henshall, D. C. (2020). A systems approach delivers a functional microRNA catalog and expanded targets for seizure suppression in temporal lobe epilepsy. *Proceedings of the National Academy of Sciences of the United States of America*, *117*(27), 15977–15988. https://doi.org/10.1073/pnas.1919313117
